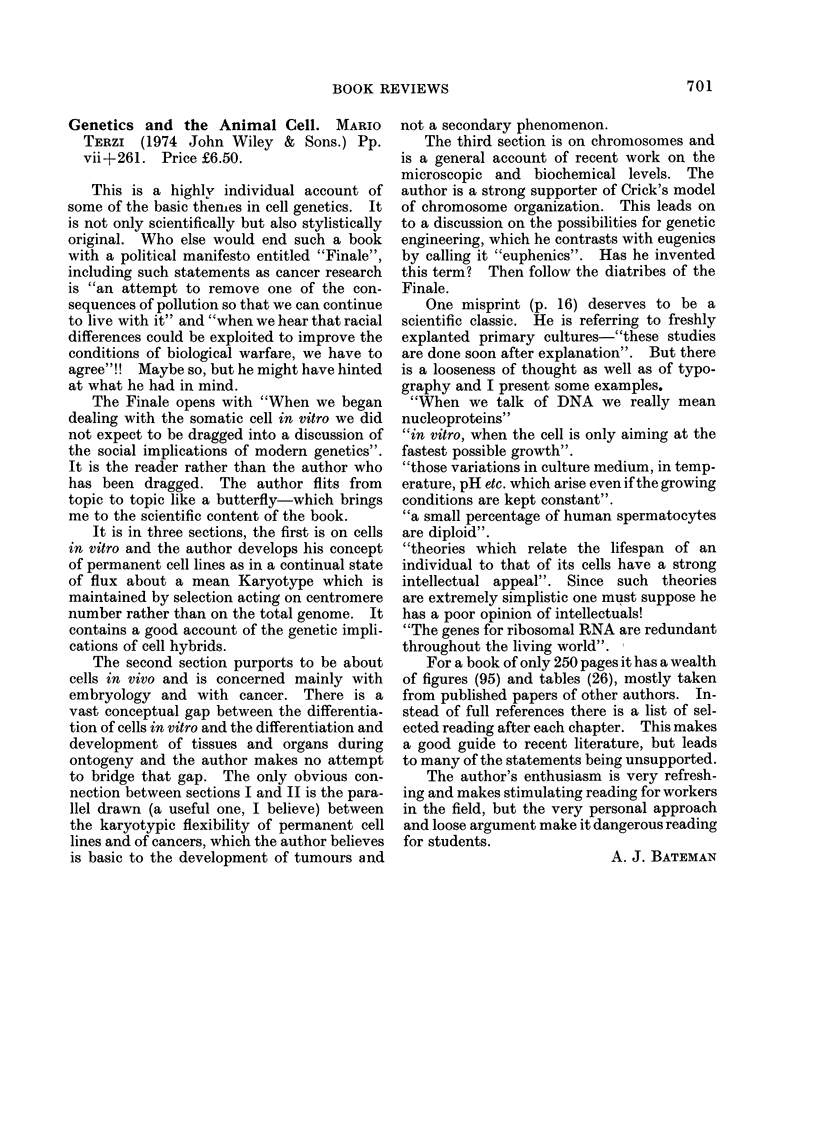# Genetics and the Animal Cell

**Published:** 1975-06

**Authors:** A. J. Bateman


**Genetics and the Animal Cell.** Mario Terzi (1974 John Wiley & Sons.) Pp. vii+261. Price £6.50.

This is a highly individual account of some of the basic themes in cell genetics. It is not only scientifically but also stylistically original. Who else would end such a book with a political manifesto entitled "Finale", including such statements as cancer research is "an attempt to remove one of the consequences of pollution so that we can continue to live with it" and "when we hear that racial differences could be exploited to improve the conditions of biological warfare, we have to agree"!! Maybe so, but he might have hinted at what he had in mind.

The Finale opens with "When we began dealing with the somatic cell *in vitro* we did not expect to be dragged into a discussion of the social implications of modern genetics". It is the reader rather than the author who has been dragged. The author flits from topic to topic like a butterfly—which brings me to the scientific content of the book.

It is in three sections, the first is on cells *in vitro* and the author develops his concept of permanent cell lines as in a continual state of flux about a mean Karyotype which is maintained by selection acting on centromere number rather than on the total genome. It contains a good account of the genetic implications of cell hybrids.

The second section purports to be about cells *in vivo* and is concerned mainly with embryology and with cancer. There is a vast conceptual gap between the differentiation of cells *in vitro* and the differentiation and development of tissues and organs during ontogeny and the author makes no attempt to bridge that gap. The only obvious connection between sections I and II is the parallel drawn (a useful one, I believe) between the karyotypic flexibility of permanent cell lines and of cancers, which the author believes is basic to the development of tumours and not a secondary phenomenon.

The third section is on chromosomes and is a general account of recent work on the microscopic and biochemical levels. The author is a strong supporter of Crick's model of chromosome organization. This leads on to a discussion on the possibilities for genetic engineering, which he contrasts with eugenics by calling it "euphenics". Has he invented this term? Then follow the diatribes of the Finale.

One misprint (p. 16) deserves to be a scientific classic. He is referring to freshly explanted primary cultures—"these studies are done soon after explanation". But there is a looseness of thought as well as of typography and I present some examples.

"When we talk of DNA we really mean nucleoproteins"

"*in vitro*, when the cell is only aiming at the fastest possible growth".

"those variations in culture medium, in temperature, pH *etc.* which arise even if the growing conditions are kept constant".

"a small percentage of human spermatocytes are diploid".

"theories which relate the lifespan of an individual to that of its cells have a strong intellectual appeal". Since such theories are extremely simplistic one must suppose he has a poor opinion of intellectuals!

"The genes for ribosomal RNA are redundant throughout the living world".

For a book of only 250 pages it has a wealth of figures (95) and tables (26), mostly taken from published papers of other authors. Instead of full references there is a list of selected reading after each chapter. This makes a good guide to recent literature, but leads to many of the statements being unsupported.

The author's enthusiasm is very refreshing and makes stimulating reading for workers in the field, but the very personal approach and loose argument make it dangerous reading for students.

A. J. Bateman